# Temporal trends of the incidence rate of syphilis during pregnancy and congenital syphilis in São Paulo, Brazil, 2011-2023

**DOI:** 10.1590/S2237-96222024v33e2024637.en

**Published:** 2025-01-27

**Authors:** Beatriz Poddis Busquim e Silva, Fernanda Penido Matozinhos, Rafaela Siqueira Costa Schreck, Fernanda Marçal Ferreira, Camilla Pontes Bezerra, Bárbara Tideman Sartório Camargo, Thales Philipe Rodrigues da Silva

**Affiliations:** 1 Universidade Federal de São Paulo, , São Paulo, SP, Brazil; 2 Universidade Federal de Minas Gerais, Departamento de Enfermagem Materno-Infantil e Saúde Pública, Belo Horizonte, MG, Brazil; 3 Universidade de São Paulo, Departamento de Enfermagem Materno-Infantil e Psiquiátrica, São Paulo, SP, Brasil; 4 Universidade Federal de São Paulo, Departamento de Enfermagem na Saúde da Mulher, São Paulo, SP, Brasil; 5 Universidade Federal de São Paulo, Programa de Pós-graduação em Enfermagem, São Paulo, SP, Brasil

**Keywords:** Sífilis, Sífilis Congénita, Embarazadas, Vigilancia Epidemiológica, Enfermedades de Transmisión Sexual, Syphilis, Syphilis, Congenital, Pregnant Women, Epidemiological Monitoring, Sexually Transmitted Diseases

## Abstract

**Objective:**

To analyze trends of syphilis during pregnancy and congenital syphilis, based on reported cases in São Paulo, Brazil, from 2011 to 2023.

**Methods:**

Ecological time series study, based on data from Notifiable Health Conditions Information System records. The Prais-Winsten method was used to verify trends.

**Results:**

125,776 cases of syphilis during pregnancy and 42,418 cases of congenital syphilis were reported. Average annual percentage change (95%CI) in the syphilis during pregnancy incidence rate was 18.68 (95%CI 16.57; 20.84), pvalue < 0.001; while for congenital syphilis it was 11.99 (95%CI 7.00; 17.22), p-value < 0.001.

**Conclusion:**

Incidence rates of syphilis during pregnancy and congenital syphilis showed significant increasing trends, which may be related to the increase in the former during the diagnosis period (1st trimester), demonstrating increased testing among pregnant women, clinical classification at the time of diagnosis (latent syphilis), as well as inefficiency of adequate treatment when diagnosis is late.

## Study contributions

**Main results:**

Rising incidence rate trends were found for syphilis during pregnancy (SP) and congenital syphilis (CS) in the state of São Paulo, as well as a rising trend in diagno-sis of SP in the 1st trimester and clinical classification of latent syphilis.

**Implications for services:**

Analysis of SP and CS enabled a better understanding of the general panorama of these cases, and will help to identify gaps in the healthcare system network, contrib-uting to better development of syphilis prevention strategies.

**Perspectives:**

Based on the results found, we suggest continuing education for primary health care professionals, to identify infection, provide correct treatment and support edu-cational actions to prevent syphilis during pregnancy.

## RESUMEN

Objetivo: Analizar la prevalencia de sífilis gestacional y congénita, a partir de las notificaciones en San Pablo, Brasil, de 2011 a 2023.

Estudio de series de tiempo ecológicas, considerando el estado de San Pablo como unidad de análisis, a partir de registros del Sistema de Información de Enfermedades de Declaración Obligatoria. Para comprobar las tendencias se utilizó el método Prais-Winsten.

Se notificaron 125.776 casos de sífilis gestacional y 42.418 casos de sífilis congénita. La variación porcentual anual (IC95%) de la tasa de incidencia de sífilis gestacional fue de 18,68 (IC95% 16,57; 20,84), p-valor < 0,001; mientras que la de sífilis congénita fue de 11,99 (IC95% 7,00; 17,22), p-valor < 0,001.

El análisis de tendencia temporal mostró que las tasas de incidencia de sífilis gestacional y sífilis congénita evidenciaron tendencias crecientes significativas, que pueden relacionarse con el aumento en las proporciones de sífilis gestacional para el período gestacional del diagnóstico (primer trimestre), evidenciando un aumento en las pruebas entre mujeres embarazadas, la clasificación clínica al momento del diagnóstico (sífilis latente), así como la ineficacia del tratamiento adecuado con diagnósticos tardíos.

## INTRODUCTION

Sexually transmitted infections (STIs) are a serious public health problem and have both social and economic impact. According to World Health Organization data, syphilis affects around 6 million people every year and, among pregnant women, it can have serious repercussions for newborn babies, when not treated adequately._1_ According to data from the Ministry of Health 2022 Epidemiological Bulletin, in Brazil, 167,523 cases of syphilis were reported on the Notifiable Health Conditions Information System (*Sistema de Informação de Agravos de Notificação* - SINAN) and incidence rates in pregnant women showed an upward trend, although at a slower rate, especially in the last four years._2_

In 2021, 27.1 cases of syphilis during pregnancy (SP) were registered per 1,000 live births in Brazil, with the Southeast region of the country coming in first place in terms of reported cases, with 44.6% of cases; the state of São Paulo recorded the second highest incidence rate in the region, with 27.1 new cases per 1,000 live births. In cases of congenital syphilis (CS), despite a decrease in incidence rates until 2018, between 2020 and 2021, a 14.6% increase was seen. In 2021, the Southeast region had the highest CS incidence rate, with 11.2 new cases per 1,000 live births. In the context of the Southeast region of Brazil, the state of São Paulo recorded the third highest CS incidence rate, with 7.1 new cases per 1,000 live births._2_

SP can be treated based on diagnosis, which can be done through rapid tests and serology exams in primary health care services. Treatment involves administration of a few doses (depending on the clinical diagnosis of the infection) of benzathine penicillin, intramuscularly, with an interval of one week between each injection. The monitoring of a pregnant woman, previously diagnosed with syphilis, begins with monthly VDRL test titration. In order to be considered adequately treated, the titer must drop twice in a three-month period, or four times in a six-month period._3_

When untreated or inadequately treated, the main repercussions of SP can be spontaneous abortion, early fetal death, stillbirth, neonatal death, premature labor, delivery and birth, low birth weight and CS._4_ It is known that half of the pregnancies of women with syphilis acquired during pregnancy can lead to adverse outcomes, one of the main ones being stillbirth. Among the reasons for stillbirth, stillbirth caused by syphilis infection is the second highest in the world, and global strategies have already been launched to reduce the number of unfavorable outcomes in SP cases by 2030._1_ In the event of vertical transmission, newborns with CS should receive treatment for ten days with crystalline or procaine penicillin._3_

Despite availability of a treatment regimen for pregnant women and their partners, the SP and CS numbers remain high. Studies show several causes for this problem that still persists in Brazil, such as ineffective control of partner treatment, lack of adequate information for pregnant women about the impacts of SP on them and the newborn baby, situations of social vulnerability that prevent adequate prenatal care, and it being difficult for health professionals to access people with greater vulnerability to syphilis infections._5_

Primary health care plays a fundamental role in this scenario, as, based on disease indicators, such as CS and SP, it is possible to verify the effectiveness of the measures in use, and in this case, it can therefore be an indicator of the quality of prenatal care. The importance of maintaining updated strategies stands out, in accordance with the most recent discussions, for better monitoring of cases and adequate access to treatment by pregnant women and their partners, aiming to reduce the numbers of SP cases and, consequently, the numbers of CS cases as well._6_

In this context, this study’s main guiding question was as follows what is the trend in SP and CS notifications in the state of São Paulo? There is a need to gain better understanding of the epidemiological profile of SP and CS in locations such as São Paulo, since studies on São Paulo state data are still scarce in the literature and are generally focused on specific municipalities. Furthermore, a better understanding of the general panorama of these cases helps to identify gaps in the health system network and contributes to the better development of strategies for the prevention and treatment of syphilis, mainly to reduce and control vertical transmission in confirmed cases.

As such, the objective of this study was to analyze trends of syphilis during pregnancy and congenital syphilis, based on reported cases in São Paulo state, Brazil, from 2011 to 2023.

## METHODS

This was an epidemiological time series study with an ecological design, carried out with SP and CS cases reported on the SINAN system, from 2011 to 2023, considering the state of São Paulo as the unit of analysis.

The state is made up of 645 municipalities, distributed over an area of 248,219.485 km^2^. Its population was 44,411,238 inhabitants in 2022, making it Brazil’s most populous Federative Unit. The state is divided into 17 Regional Health Departments, and there are currently discussions as to the Health Regionalization Master Plan, with the aim of organizing and planning access to health services and the flow of available services.^7,8^

Data on SP and CS cases were initially accessed by extracting them via the Information Technology Department of the Brazilian National Health System (DATASUS), and were then tabulated using TABNET. They were then extracted from the following website http://indicadoressifilis.aids.gov.br/, using the TABNET filter for the “state of São Paulo”. The data were exported on January 31, 2024.⁹

The indicators considered for this study were the SP and CS incidence rates in São Paulo. However, it should be noted that the year 2023 was excluded with regard to SP and CS incidence rates, as these indicators were not available for that year. The SINAN adopts the following method to calculate the SP and CS incidence rates: for the SP incidence rate, it takes the number of reported or confirmed cases in pregnant women in São Paulo, divided by the number of live births in the state, multiplied by 1000; in turn, for the CS incidence rate, it takes the number of new cases of congenital syphilis per year, divided by the number of live births in the state in the same year, multiplied by 1000. 

The variables present on the SP and CS notification forms were also analyzed, using the absolute and relative frequencies of case notifications in the state, according to the specified characteristics and categories. The variables analyzed are specified below. 

Sociodemographic characteristics (maternal age group, in years); child’s age; maternal schooling; and maternal race/skin color. Clinical characteristics (clinical classification of SP; gestational age when diagnosed; time of SP diagnosis; and maternal treatment regimen).

Data were analyzed using the STATA version 16.0 statistical package. The variables on SP and CS were described using absolute and relative frequencies.

We used the autoregressive models proposed by Prais-Winsten to analyze the temporal trends, whereby the dependent variables were the incidence rates and the proportions of sociodemographic and clinical characteristics of SP and CS; and the independent variables were the years of the study (2011 to 2022 for the incidence rates and 2011 to 2023 for the remaining analyses). The Prais-Winsten regression model was chosen due to the possibility of correcting serial autocorrelation arising from time series. In order to correct the heterogeneity of residual variance, the outcome was transformed using a logarithmic scale._10_

Subsequently, we calculated annual percentage change (APC). The following formula was used to calculate APC, as per a study by Antunes and Cardoso:_10_ APC = (-1+10[^b1^]*100%), *where* b1 refers to the Prais-Winsten regression angular coefficient (beta)._10_

We also calculated the 95% confidence intervals (95%CI) of the APC measurements, using the following formula: lower 95%CI (-1+10[^b1-t^*^e^]*100%) and upper 95%CI (-1+10[^b1+t^*^e^ ]*100%).

The regression results were interpreted as follows: rising trend, when the regression angular coefficient was positive and had a p-value less than 0.05; falling trend, when the regression angular coefficient was negative and the p-value was less than 0.05; or stationary trend, when the p-value was greater than 0.05._10_

As these were non-nominal public data, available via DATASUS, approval of the study project by a Research Ethics Committee was not required.

## RESULTS

**Figure 1:**
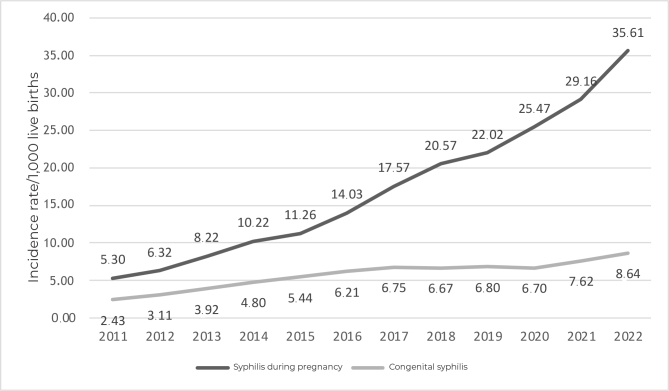
Incidence rates of syphilis during pregnancy and congenital syphilis, São Paulo, Brazil, 2011-2022

In the period from 2011 to 2022, 125,776 cases of SP and 42,418 cases of CS were identified in São Paulo. During this period, an increase in the incidence rates of SP and CS was seen (Figure 1). 

Table 1 shows that among the reported cases of SP, 56.43% (n = 70,980) of women were in the 20-29 age group; 29.52% (n = 37,132) had completed high school and 20.99% (n = 26,397) had an unknown schooling level; and 41.62% (n = 52,345) self-reported being of White race/skin color, while 41.07% (n = 51,662) self-reported being of mixed race/skin color.

**Table 1:** Absolute and relative distribution of sociodemographic characteristics of cases of syphilis during pregnancy and congenital syphilis, São Paulo, Brazil, 2011-2023

**Sociodemographic characteristics**	**n**	**%**
**Syphilis during pregnancy**		
**Maternal age (years) (n = 125,775)**		
10-14	993	0.79
15-19	26,719	21.24
20-29	70,980	56.43
30-39	24,287	19.31
≥ 40	2,796	2.22
**Maternal schooling (n = 125,776)**		
Illiterate	310	0.25
Incomplete 1^st^ to 4^th^ grade	3,433	2.73
Complete 4^th^ grade	3,258	2.59
Incomplete 5^th^ to 8^th^ grade	16,385	13.03
Complete elementary education	12,634	10.04
Incomplete high school education	21,913	17.42
Complete high school education	37,132	29.52
Incomplete higher education	2,213	1.76
Complete higher education	2,101	1.67
Unknown	26,397	20.99
**Race/skin color (n = 125,776)**		
White	52,345	41.62
Black	14,658	11.65
Asian	730	0.58
Mixed race	51,662	41.07
Indigenous	263	0.21
Unknown	6,118	4.86
**Congenital syphilis**		
**Child’s age (n = 38,417)**		
Under 7 days	37,344	97.21
7-27 days	420	1.09
28-364 days	561	1.46
1 YEAR	55	0.14
2 A 4 YEARS	26	0.07
5 a 12 years	11	0.03
**Maternal age (years) (n = 42,418)**		
10-14	232	0.55
15-19	8,078	19.04
20-29	23,726	55.93
30-39	8,808	20.76
≥ 4	1,007	2.37
Unknown	567	1.34
**Maternal schooling (n = 42,418)**		
Illiterate	261	0.62
Incomplete 1^st^ to 4^th^ grade	1,350	3.18
Complete 4^th^ grade	977	2.30
Incomplete 5^th^ to 8^th^ grade	6,000	14.14
Complete elementary education	4,549	10.72
Incomplete high school education	6,060	14.29
Complete high school education	9,844	23.21
Incomplete higher education	528	1.24
Complete higher education	571	1.35
Not applicable	216	0.51
Unknown	12,062	28.44
**Maternal race/skin color (n = 42,418)**		
White	17,300	40.78
Black	3,485	8.22
Asian	105	0.25
Mixed race	18,027	42.50
Indigenous	70	0.17
Unknown	3,431	8.09
**Final diagnosis (n = 42,418)**		
Early congenital syphilis	38,110	89.84
Late congenital syphilis	38	0.09
Miscarriage due to syphilis	2,703	6.37
Stillbirth due to syphilis	1,567	3.69

Among CS notifications, 97.21% (n = 37,344) of babies were less than 7 days old; 55.29% (n = 23,726) were born to mothers aged between 20 and 29 years; 23.21% (n = 9,844) were born to mothers had completed high school education, while schooling level was unknown for 28.44% (n = 12,062) of them; 42.50% (n = 18,027) self-reported maternal mixed race/skin color, while 40.78% (n = 17,300) self-reported White race skin/color. Among the diagnoses, 89.84% (n = 38,110) were recent CS cases.

Regarding clinical characteristics, 58.05% (n = 73,010) of SP cases were latent and 53.74% (n = 67,594) of SP cases were identified in the 1st trimester of pregnancy. In turn, 60.65% (n = 25,727) of CS cases were diagnosed during the mother’s prenatal care, while 32.78% (n = 13,906) were diagnosed at the time of birth or curettage; 49.50% (n = 20,996) underwent inadequate treatment and 34.10% (n = 14,463) did not undergo any treatment (Table 2).

**Table 2:** Absolute and relative distribution of the clinical characteristics of detection of syphilis during pregnancy and congenital syphilis, São Paulo, Brazil, 2011-2023

**Clinical characteristics**	**n**	**%**
**Syphilis during pregnancy (n = 125,776)**		
**Clinical classification**	24,890	19.79
Primary syphilis	4,502	3.58
Secondary syphilis	7,346	5.84
Tertiary syphilis	73,010	58.05
Latent syphilis	16,028	12.74
Unknown	16.028	12,74
1^st^ trimester	67,594	53.74
2^nd^ trimester	30,629	24.35
3^rd^ trimester	23,972	19.06
Unknown	3,581	2.85
**Congenital syphilis (n = 42,418)**		
**Time at which syphilis during pregnancy diagnosed**		
Durante o prenatal care	25,727	60.65
At delivery/curettage	13,906	32.78
After delivery	1,542	3.64
Not performed	253	0.60
Unknown	990	2.33
**Maternal treatment regimen**		
Adequate	2,759	6.50
Inadequate	20,996	49.50
Not performed	14,463	34.10
Unknown	4,200	9.90

Table 3 shows the proportions of SP and CS, according to sociodemographic and clinical variables. It can be seen that the highest number of reported SP and CS cases was 2022. In relation to sociodemographic data, maternal age of 20-29 years predominated during all years of analysis. Regarding the period of diagnosis, diagnosis continued to be higher during the 1^st^ trimester, for SP. Regarding diagnostic clinical characteristics, the proportions were higher for latent syphilis from the beginning in SP cases, and recent CS for CS cases. Regarding maternal treatment regimen in CS cases, inadequate treatment predominated throughout the period.

**Table 3:** Proportion of syphilis during pregnancy and congenital syphilis according to sociodemographic and clinical variables, by year of notification, São Paulo, Brazil, 2011-2023

**Sociodemographic and clinical variables**	**Year**												
	**2011**	**2012**	**2013**	**2014**	**2015**	**2016**	**2017**	**2018**	**2019**	**2020**	**2021**	**2022**	**2023**
Case of syphilis during pregnancy													
	n = 3,234	n = 3,899	n = 5,022	n = 6,392	n = 7,138	n = 8,437	n = 10,747	n = 12,467	n = 12,841	n = 14,067	n = 15,317	n = 18,702	n = 7,513
**Proportion of cases of syphilis during pregnancy by period of diagnosis**													
1^st^ trimester	35.56	37.42	39.53	41.96	44.89	49.47	53.24	52.39	53.37	58.51	60.53	61.58	64.09
2^nd^ trimester	32.90	33.24	34.93	32.81	32.56	30.45	27.37	24.27	23.51	20.30	18.92	18.67	17.21
3^Rd^ trimester	28.01	26.08	22.66	22.43	19.91	17.71	17.25	20.12	19.54	18.48	17.99	16.86	15.75
Unknown	3.53	3.26	2.89	2.80	2.65	2.37	2.14	3.22	3.58	2.71	2.57	2.89	2.95
**Proportion of cases of syphilis during pregnancy by age group (years) of the pregnant woman at the time of diagnosis**													
10-14	0.87	1.03	1.06	0.97	0.87	1.02	0.81	0.92	0.62	0.84	0.65	0.66	0.53
15-19	18.34	20.24	21.55	22.43	23.26	23.56	23.68	22.53	22.40	22.26	20.51	18.18	16.92
20-29	49.88	49.60	51.19	51.28	52.28	52.74	53.44	54.53	56.86	57.69	59.82	61.87	62.70
30-39	26.50	25.60	23.87	22.48	21.38	20.53	20.01	19.85	18.10	17.20	17.12	17.21	17.66
≥ 40	4.42	3.54	2.33	2.83	2.21	2.15	2.07	2.17	2.02	2.01	1.90	2.07	2.18
**Proportion of cases of syphilis during pregnancy, by clinical classification of syphilis during pregnancy at the time of diagnosis**													
Primary syphilis	30.12	30.60	26.09	24.78	23.34	22.00	20.94	19.98	16.39	16.19	17.70	17.29	16.49
Secondary syphilis	7.42	6.36	6.39	6.65	5.41	4.39	3.68	3.13	3.03	2.35	2.37	2.37	2.68
Tertiary syphilis	10.79	9.05	12.13	11.14	9.53	7.35	6.35	5.57	4.22	4.17	3.96	3.67	2.99
Latent syphilis	32.50	37.47	39.11	42.37	45.15	50.87	55.28	56.63	62.88	68.66	65.55	66.88	66.92
Unknown	19.17	16.52	16.29	15.07	16.57	15.40	13.76	14.69	13.47	8.63	10.42	9.79	10.91
**Cases of congenital syphilis**													
	n = 1,485	n = 1,919	n = 2,394	n = 3,006	n = 3,450	n = 3,732	n = 4,131	n = 4,041	n = 3,967	n = 3,699	n = 4,004	n = 4,536	n = 2,054
**Proportion of final diagnosis**													
Early congenital syphilis	92.59	91.45	91.85	89.79	90.52	91.56	90.83	88.37	88.73	88.54	88.54	88.93	89.82
Late congenital syphilis	0.13	0.21	0.00	0.10	0.12	0.05	0.15	0.02	0.18	0.03	0.02	0.13	0.05
Miscarriage due to syphilis	4.38	4.64	4.76	5.12	5.45	4.88	5.52	7.60	7.71	7.79	7.52	7.67	6.48
Stillbirth due to syphilis	2.90	3.70	3.38	4.99	3.91	3.51	3.51	4.01	3.38	3.65	3.92	3.26	3.65
**Proportion of cases of congenital syphilis according to maternal treatment regimen**													
Adequate	3.77	2.92	3.26	2.69	4.09	4.85	4.94	7.13	6.78	8.27	10.06	11.02	9.54
Inadequate	52.32	51.38	51.38	55.59	52.64	55.68	53.84	48.82	46.99	44.93	42.06	44.93	48.34
Not performed	35.96	38.82	34.00	32.90	31.71	28.91	29.61	33.18	35.44	34.31	41.23	36.18	32.96
Unknown	7.95	6.88	11.36	8.82	11.57	10.56	11.62	10.86	10.79	12.49	6.64	7.87	9.15

Table 4 shows the results of the trend analysis, as well as the APC of SP and CS incidence rates and the proportions of SP and CS cases, according to notification sociodemographic and clinical variables. A rising trend was found for the SP incidence rates (2011-2022) and CS incidence rates (2011-2022). Rising trends were also found for all the following variables: SP cases when syphilis was diagnosed in the 1st trimester of pregnancy (APC = 5.10; 95%CI 4.20; 6.00); SP cases when the pregnant women were in the 20-29 age group at the time of syphilis diagnosis (APC = 1.98%; 95%CI 1.54; 2.42); mixed race/skin color at the time of syphilis diagnosis (APC = 2.05%; 95%CI 1.68; 2.41); clinical classification of latent SP at the time of syphilis diagnosis (APC = 6.33%; 95%CI 4.41; 8.30); and inadequate maternal treatment regimen in cases of CS (APC = 12.42%; 95%CI 9.17; 15.76).

**Table 4:** Trend and variation in (%) and annual average with 95% confidence intervals (95%CI) of the incidence rate of syphilis during pregnancy and congenital syphilis and of the proportions of cases of syphilis during pregnancy and congenital syphilis, according to notification sociodemographic and clinical variables, São Paulo, Brazil, 2011-2023

**Demographic and clinical variables**	**% (95%CI)**	**p-value**	**Trend**
**Incidence rate of syphilis during pregnancy (2011-2022)**	**18.68 (16.57; 20.84)**	**< 0.001**	**Rising**
**Incidence rate of congen-ital syphilis (2011-2022)**	**11.99 (7.00; 17.22)**	**< 0.001**	**Rising**
**Cases of syphilis during pregnancy, by gestational age at diagnosis**			
1^st^ trimester	5.10 (4.20; 6.00)	< 0.001	Rising
2^nd^ trimester	-5.62 (-7.42; -3.78)	< 0.001	Falling
3^rd^ trimester	-4.20 (-6.15; -2.22)	0.001	Falling
Gestational age	-0.87 (-3.93; 2.28)	0.554	Stationary
unknown			
**Cases of syphilis during pregnancy, by age group (years) of the pregnant woman at the time of diagnosis**			
10-14	-4.53 (-5.85;-3.19)	< 0.001	Falling
15-19	-0.67 (-3.96; 2.73)	0.670	Stationary
20-29	1.98 (1.54; 2.42)	< 0.001	Rising
30-39	-3.47 (-4.62; -2.31)	< 0.001	Falling
≥ 40	-5.00 (-8.29; -1.58)	0.009	Falling
**Cases of syphilis during pregnancy, by race/skin color, at the time of diagnosis**			
White	-1.28 (-1.55; -1.01)	< 0.001	Falling
Black	-0.56 (-1.46; 0.34)	0.203	Stationary
Asian	-4.05 (-6.83; -1.19)	0.011	Falling
Mixed race	2.05 (1.68; 2.41)	< 0.001	Rising
Indigenous	-12.92 (-19.05; -6.34)	0.002	Falling
Unknown	-4.25 (-9.14; 0.90)	0.099	Stationary
**Cases of syphilis during pregnancy, by clinical classification of syphilis during pregnancy at the time of diagnosis**			
Primary syphilis	-5.25 (-6.74; -3.73)	< 0.001	Falling
Secondary syphilis	-9.15 (-12.38; -5.80)	< 0.001	Falling
Tertiary syphilis	-10.88 (-13.61; -8.06)	< 0.001	Falling
Latent syphilis	6.33 (4.41; 8.30)	< 0.001	Rising
Diagnosis unknown	-5.30 (-7.10;- 3.46)	< 0.001	Falling
**Cases of congenital syphilis according to maternal treatment regimen**			
Adequate	12.42 (9.17; 15.76)	< 0.001	Rising
Inadequate	-1.21 (-2.73 0.35)	0.118	Stationary
Not performed	-0.13 (-2.45; 2.25)	0.910	Stationary
Unknown	0.24 (-3.63; 4.26)	0.898	Stationary

A falling trend was found for all the following variables: SP cases when syphilis was diagnosed in the 2^nd^ trimester (APC = -5.62%; 95%CI 7.42; -3.78) and 3^rd^ trimester of pregnancy (APC = -4.20%; 95%CI -6.15; -2.22); SP for pregnant women who were in the 10-14 age group (APC = -4.53; 95%CI -5.85; -3.19), the 30-39 age group (APC = -3.47%; 95%CI -4.62; -2.31) and the 40 or over age group (APC = -5.00%; 95%CI -8.29; -1.58) at the time of syphilis diagnosis; White (APC = -1.28%; 95%CI -1.55; -1.01), Asian (APC = -4.05; 95%CI -6.83; -1.19) and Indigenous (APC = -12.92%; 95%CI -19.05; -6.34) race/skin color at the time of syphilis diagnosis; clinical classification of SP as primary (APC = -5.25%; 95%CI -6.74; -3.73), secondary (APC = - 9.15; 95%CI -12.38%; -5.80) and tertiary (APC = -10.88%; 95%CI -13.61%; -8.06) at the time of syphilis diagnosis (Table 4).

## DISCUSSION

After analyzing the results found in this study, a rising trend could be seen in the incidence rates of SP and CS in the state of São Paulo. The rising trends in the proportions of SP for the gestational period of diagnosis (1^st^ period) and clinical classification at the time of diagnosis (latent syphilis) stood out, as did the proportion of CS cases according to adequate maternal treatment regimen. In relation to sociodemographic characteristics, an increase was observed for those aged 20 to 29 years and those of self-reported mixed race/skin color.

According to Brazilian Institute of Geography and Statistics (*Instituto Brasileiro de Geografia e Estatística* - IBGE) data for the year 2022, the female population of São Paulo is characterized by prevalence of the 30-39 age group and White self-declared race/skin color (13,768,294), followed by mixed race (6,001,139). Data from this study demonstrated a higher prevalence of SP cases in pregnant women between 20 and 29 years old and in mixed race women, these being results that differ from the characterization of the female population in São Paulo. Studies indicate that women of Black and mixed race/skin color suffer more significantly from social inequalities and, therefore, have less access to health education, prevention and adequate treatment._11_

Regarding the race/skin color profile of pregnant women diagnosed with SP, it was found that the highest proportion was for White women, while the highest proportion for CS was among mixed race women. Incidence of CS results from non-diagnosis in a timely manner, which does not allow for correct treatment to prevent intrauterine transmission._12_ This result may be associated with the fact that Black and mixed race women in Brazil are usually part of more socially vulnerable populations, living in regions where coverage of primary health care, for example, is not as effective, making access to health services more complex. It must also be recognized that there is a limit to the effectiveness of Brazilian public policies for maternal health when it comes to addressing ethnic-racial inequities, which is reflected in indicators that reveal obstetric racism._13_ In this sense, these women are more vulnerable to CS, due to racism and its manifestations, as a structural social determinant that imposes barriers to access to timely diagnosis and treatment.

Furthermore, this scenario was worsened by the COVID-19 pandemic, which contributed to the increase in health inequities, shifting the focus of health actions to controlling the spread of coronavirus. This rearrangement of health service organization compromised access of certain population groups to the health care network for other specific cases, including access to prenatal care._12_ Notwithstanding, in places where CS rates are higher, greater investments need to be made in order to reduce or eliminate vertical transmission rates._14_

Despite the rise in the results regarding inadequate treatment or non-treatment during prenatal care, the proportion of CS with an adequate maternal treatment regimen showed a rising trend, which indicates that there was an increase, over the years, in adherence to correct treatment among pregnant women diagnosed with syphilis; however, there is a gap between the moment treatment is performed and child delivery, such as reinfection without screening. The gold standard for treatment for SP is intramuscular (IM) administration of benzathine penicillin G, which is provided by primary care. According to the Ministry of Health, the treatment regimen for pregnant women with syphilis depends on the clinical classification of the disease, with a single dose of 2.4 million units IM for primary, secondary and recent latent syphilis; and three doses of 2.4 million units IM, one week apart, for tertiary or late latent syphilis._3_ In this sense, primary health care has a fundamental role in controlling the disease and offering treatment, as it is the gateway for people to access the health service, the place for adequate treatment and the channel of communication for educational actions for communicable disease prevention._15_

Our study showed a significant rising trend for SP proportions in clinical classification as latent syphilis at the time of diagnosis. It is known that the more recent the syphilis infection during pregnancy, the more it is harmful for the baby, but also that the likelihood of effective treatment is greater. One of the challenges in diagnosing latent syphilis is not knowing when the infection occurred, and also not knowing how long the disease has lasted, as it is generally asymptomatic and can only be visualized by means of treponemal tests. In view of this, the importance of testing during prenatal care is clear, as recommended by the Ministry of Health: testing in the first trimester, in the third trimester and before birth. Through testing, it is possible to identify pregnant women who need early treatment or other referrals, to confirm diagnosis and begin treatment._3_ Another important point to be considered is the role of primary health care in approaching people who plan to become pregnant, with testing for syphilis being recommended, which would impact the incidence of SP, since it would be possible to treat these women before they conceive._16_ However, it must be recognized that planned pregnancy is a challenge in Brazil, for reasons such as insufficient knowledge about preconception preparation and unavailability of conception care in health service routines._17_

In 2020, following enactment of São Paulo City Health Department Ordinance No. 120, on March 11, a new name was given to the Health Program for Pregnant Women and Newborns in the Municipality of São Paulo, i.e. the *Mãe Paulistana* (São Paulo Mother) Program. As part of the Program, health services must follow certain criteria during prenatal care in order to receive certification. These include early recruitment of pregnant women (up to 12 weeks of gestation) and the percentage of rapid testing for syphilis during the first prenatal care consultation at the primary health center, which ends up becoming an incentive for services to establish appropriate conduct in screening for syphilis during pregnancy. Pregnant women also have some criteria to follow in order to receive the newborn’s basic layette, including three syphilis tests and three HIV tests, which allows for early detection and the possibility of timely treatment._18_

Currently, partner treatment is not taken into account when defining adequate treatment after diagnosis. However, partner testing and treatment of positive cases helps to avoid reinfections and the likelihood of transmission to the fetus. In places that do not take into account adequate partner treatment, fetus transmission rates also tend to be higher, precisely due to the loss of control due to reinfections and the likelihood of maternal treatment failure._19_

Across the country, Brazilian National Health System (*Sistema Único de Saúde* - SUS) coverage is over 80%, and the strategies proposed by the Ministry of Health for syphilis prevention, diagnosis and treatment, during prenatal care, are well founded and easy to apply. However, the results of this study show figures that are not yet in line with what is recommended by national and international health institutions to control this infection.

It is noteworthy that screening for STIs, such as syphilis, is an important indicator for evaluating the quality of prenatal care. Therefore, reducing SP and CS cases must be approached as a public health issue, considering that there is an effective and rapid form of screening, treatment available in most locations and sufficient materials for educating the population about prevention.^3,20^

Among the main challenges found in primary health care, lack of information, both on the part of health professionals and health service users, is among the main reasons for precarious care in relation to syphilis. Furthermore, difficulty in accessing service users who find themselves in groups most vulnerable to STIs, such as syphilis, also deserves to be highlighted, as it is these pregnant women who need greater equity in accessing the services offered by the health care network._20_

As a limitation, it can be highlighted that the study was based on passive notification of identified cases of SP and CS in the state of São Paulo, which results in it being difficult to make assertive analysis, as there may be cases of underreporting, which are not included in the statistics. Furthermore, the results may be affected by periodic updating of the data, which also interferes with analysis, depending on the access date. Notwithstanding, the data presented and the discussions proposed by the study contribute to identification and analysis of socio-structural conditions related to SP and CS, helping to propose strategies to address these infections.

The temporal trend analysis showed that the incidence rates of SP and CS had significant rising trends, which may be related to the clinical classification of syphilis and the inefficiency of adequate treatment with late diagnoses. Syphilis is an STI with easy access to testing and identification of infection, availability of adequate and timely treatment, although there are currently still a large number of cases. Therefore, we suggest the continuing education of health professionals, mainly from primary health care, to identify infection, provide correct treatment and support educational actions to prevent syphilis during pregnancy and harm to newborns, focusing mainly on the prenatal care axis.

In this sense, health education and comprehensive women’s health care are part of nursing’s scope of action, so that health professionals in the sector are therefore an important category for prevention, diagnosis and care in cases of SP and CS. However, these actions must be coordinated with other primary health care professionals, so that, in a multidisciplinary way, there is control of syphilis infections during pregnancy.
