# Supplementary material for: Temporal trends of the incidence rate of syphilis during pregnancy and congenital syphilis in São Paulo, Brazil, 2011-2023
Source: Epidemiol Serv Saude. 2025 Jan 27;33:e2024637. doi: 10.1590/S2237-96222024v33e2024637.en (PMC11776069; doi:10.1590/S2237-96222024v33e2024637.en)
Supplement: Translation

# Translation: Tendência temporal da taxa de incidência de sífilis gestacional e congênita em São Paulo, 2011-2023

**DOI:** 10.1590/S2237-96222024v33e2024637.pt

**Authors:** Beatriz Poddis Busquim e Silva, Fernanda Penido Matozinhos, Rafaela Siqueira Costa Schreck, Fernanda Marçal Ferreira, Camilla Pontes Bezerra, Bárbara Tideman Sartório Camargo, Thales Philipe Rodrigues da Silva

**Affiliations:** 1 Universidade Federal de São Paulo, Programa de Residência em Enfermagem Obstétrica, São Paulo, SP, Brasil; 2 Universidade Federal de Minas Gerais, Departamento de Enfermagem Materno-Infantil e Saúde Pública, Belo Horizonte, MG, Brasil; 3 Universidade de São Paulo, Departamento de Enfermagem Materno-Infantil e Psiquiátrica, São Paulo, SP, Brasil; 4 Universidade Federal de São Paulo, Departamento de Enfermagem na Saúde da Mulher, São Paulo, SP, Brasil; 5 Universidade Federal de São Paulo, Programa de Pós-graduação em Enfermagem, São Paulo, SP, Brasil

**Keywords:** Sífilis, Sífilis Congênita, Gestantes, Monitoramento Epidemiológico, Doenças Sexualmente Transmissíveis

## Abstract

**Objetivo:**

Analisar a tendência de sífilis gestacional e congênita, a partir das notificações em São Paulo, Brasil, de 2011 a 2023.

**Métodos:**

Estudo ecológico de série temporal, considerando como unidade de análise o estado de São Paulo, a partir dos registros no Sistema de Informação de Agravos de Notificação. Empregou-se o método de Prais-Winsten para verificação de tendência.

**Resultados:**

Foram notificados 125.776 casos de sífilis gestacional e 42.418 casos de sífilis congênita. A variação percentual anual (IC_95%_) para a taxa de incidência de sífilis gestacional foi de 18,68 (IC_95%_ 16,57; 20,84), p-valor < 0,001; enquanto para a sífilis congênita foi de 11,99 (IC_95%_ 7,00; 17,22), p-valor < 0,001.

**Conclusão:**

A análise de tendência temporal evidenciou que as taxas de incidência de sífilis gestacional e sífilis congênita apresentaram tendências crescentes significativas, o que se pode relacionar ao aumento nas proporções de sífilis gestacional para o período gestacional do diagnóstico (1º trimestre), mostrando aumento na testagem entre as gestantes, a classificação clínica no momento do diagnóstico (sífilis latente), bem como a ineficiência do tratamento adequado com os diagnósticos tardios.

## Contribuições do estudo

**Principais resultados:**

Observou-se tendência crescente para taxas de incidência de sífilis gestacional (SG) e sífilis congênita (SC) no estado de São Paulo. Além da tendência crescente no diagnóstico de SG no 1º trimestre e classificação clínica de sífilis latente.

**Implicações para os serviços:**

A análise de SG e SC possibilitou melhor entendimento do panorama geral desses casos e poderá auxiliar na identificação de lacunas na rede de assistência no sistema de saúde e contribuir para melhor elabora-ção de estratégias de prevenção da sífilis.

**Perspectivas:**

Com os resultados encontrados, sugere-se capacitação continuada dos profissionais de saúde da Atenção Primária à Saúde para a identificação da infecção, tratamento correto e apoio às ações educativas de prevenção da sífilis na gestação.

## INTRODUÇÃO

As infecções sexualmente transmissíveis (IST) configuram-se como um grave problema de saúde pública e geram impacto nas esferas social e econômica da população. Segundo dados da Organização Mundial da Saúde, a sífilis atinge cerca de 6 milhões de pessoas todo ano e, na gestante, pode trazer repercussões graves para o recém-nascido (RN), quando não tratada adequadamente.^1^ Dados do Boletim Epidemiológico de 2022 do Ministério da Saúde, no Brasil, 167.523 casos de sífilis foram notificados no Sistema de Informação de Agravos de Notificação (Sinan) e as taxas de incidência em gestantes apresentou tendência crescente, mas em velocidade menor, principalmente nos últimos quatros anos.^2^

Em 2021, foram registrados 27,1 casos de sífilis gestacional (SG) a cada 1 mil nascidos vivos no Brasil, sendo a região Sudeste a primeira em relação aos casos notificados, com 44,6% dos casos, e o estado de São Paulo registrou a segunda maior taxa de incidência da região Sudeste com 27,1 novos casos a cada 1 mil nascidos vivos. Nos casos de sífilis congênita (SC), apesar de um decréscimo nas taxas de incidência até o ano de 2018, entre 2020 e 2021 observa-se aumento de 14,6%. Em 2021, a região Sudeste apresentou a maior taxa de incidência de SC, sendo 11,2 novos casos a cada 1 mil nascidos vivos. Nesse contexto da região Sudeste do Brasil, o estado de São Paulo registrou a terceira maior taxa de incidência de SC, com 7,1 novos casos a cada 1 mil nascidos vivos.^2^

A SG pode ser tratada a partir do diagnóstico, que pode ser feito por meio de testes rápidos e exames de sorologia na Atenção Primária à Saúde (APS). O tratamento é realizado com aplicação de algumas doses (a depender do diagnóstico clínico da infecção) de penicilina benzatina, intramuscular, com intervalo de uma semana entre cada administração. O acompanhamento de uma gestante, previamente diagnosticada com sífilis, acontece a partir da titulação mensal exame de VDRL mensalmente. Para que seja considerada adequadamente tratada, essa titulação deve cair em duas vezes no período de três meses ou quatro vezes em um período de seis meses.^3^

Quando não tratada ou tratada inadequadamente, as principais repercussões da SG podem ser o abortamento, óbito fetal precoce, natimortalidade, óbito neonatal, trabalho de parto, parto e nascimento prematuro, baixo peso ao nascer e SC.^4^ Sabe-se que metade das gestações de mulheres com sífilis adquirida durante a gestação pode levar a desfechos adversos, sendo um dos principais a natimortalidade. Entre os motivos da natimortalidade, a causada pela infecção por sífilis é a segunda maior no mundo e já existem estratégias globais lançadas para diminuir os números de desfechos desfavoráveis nos casos de SG até 2030.^1^ Caso haja a transmissão vertical, o RN com SC deve receber o tratamento por 10 dias com penicilina cristalina ou procaína.^3^

Apesar da disponibilidade de um esquema de tratamento para as gestantes e suas parcerias, os números de SG e SC continuam altos. Estudos mostram diversas causas para esse problema ainda persistente no Brasil, como ineficácia de controle do tratamento da parceria, falta de informações adequadas para as gestantes sobre os impactos da SG para ela e para o RN, situações de vulnerabilidade social que impedem o pré-natal adequado, difícil acesso dos profissionais de saúde a pessoas com maior vulnerabilidade para infecções por sífilis.^5^

A APS possui um papel fundamental nesse cenário, pois a partir dos indicadores de doenças, como a SC e a SG, é possível verificar a eficácia das medidas em uso e, nesse caso, podendo ser, portanto, um indicador da qualidade do pré-natal. Ressalta-se a importância da manutenção de estratégias atualizadas de acordo com as discussões mais recentes, para um melhor monitoramento dos casos e acesso adequado ao tratamento pelas gestantes e seus parceiros, visando à redução dos números de SG e, por consequência, de SC.^6^

Diante desse contexto, este estudo teve como principal questão norteadora saber qual a tendência das notificações de sífilis gestacional e congênita no estado de São Paulo? Evidencia-se a necessidade de melhor compreensão do perfil epidemiológico da SG e da SC em localidades como São Paulo, uma vez que os estudos sobre os dados do estado ainda são escassos na literatura e, geralmente, focados em municípios específicos. Ademais, o melhor entendimento do panorama geral desses casos auxilia na identificação de lacunas na rede de assistência no sistema de saúde e contribui para a melhor elaboração de estratégias de prevenção e tratamento da sífilis, principalmente para redução e controle da transmissão vertical em casos confirmados.

Sendo assim, o objetivo do estudo foi analisar a tendência temporal de sífilis gestacional e congênita, a partir de notificações no estado de São Paulo, Brasil, no período de 2011 a 2023.

## MÉTODOS

Estudo epidemiológico com delineamento ecológico, de série temporal realizado com os casos notificados de sífilis gestacional (SG) e sífilis congênita (SC) no Sinan, no período de 2011 a 2023, considerando-se como unidade de análise o estado de São Paulo.

O estado de São Paulo é composto por 645 municípios, distribuídos em uma are territorial com 248.219,485 km², e uma população de 44.411.238 habitantes para o ano de 2022, sendo o estado mais populoso do país. O estado é dividido em 17 Departamentos Regionais de Saúde e atualmente existem discussões sobre o Plano Diretor de Regionalização da Saúde, com o objetivo de organizar e planejar o acesso aos serviços de saúde e o fluxo dos serviços já disponíveis.^7,8^

Para acessar os dados dos casos de SG e SC, realizou-se a extração via Departamento de Informática do Sistema Único de Saúde (DATASUS) e a tabulação no TABNET. Os dados obtidos via DATASUS foram extraídos via sítio eletrônico (http://indicadoressifilis.aids.gov.br/), aplicando o filtro para o “estado de São Paulo”. Os dados foram exportados do DATASUS, via TABNET, em 31 de janeiro de 2024.^9^

Para este estudo, os indicadores considerados foram as taxas de incidência de SG e SC de São Paulo. Ressalta-se que, para as taxas de incidência de SG e SC, excluiu-se o ano de 2023 por ausência do indicador para o ano em questão. O Sinan adota para o cálculo das taxas de incidência de SG e SC o seguinte método: para o cálculo da taxa de incidência da SG, o número de casos notificados ou confirmados em gestantes de São Paulo dividido pelo número de recém-nascidos vivos no estado de São Paulo, multiplicado por mil (1.000); Já para a SC, o método de cálculo é o número de casos novos de sífilis congênita ocorridos no estado de São Paulo por ano, dividido pelo número de recém-nascidos vivos no estado no mesmo ano, multiplicado por mil (1.000). 

Também foram analisadas as variáveis presentes nas fichas de notificação de SG e SC, utilizando-se as frequências absoluta e relativa das notificações dos casos no estado, de acordo com as características e categorias especificadas. As variáveis analisadas incluem: Características sociodemográficas (faixa etária materna - em anos); idade da criança; escolaridade materna; e raça/cor da pele da gestante) e Características clínicas (classificação clínica da SG; idade gestacional do diagnóstico; momento do diagnóstico da sífilis materna; e esquema de tratamento materno).

Os dados foram analisados utilizando-se o Stata, versão 16.0. As variáveis sobre a SG e SC foram apresentadas por meio das frequências absoluta e relativa.

Na análise das tendências temporais, empregou-se os modelos autorregressivos proposto por Prais-Winsten, sendo as variáveis dependentes as taxas de incidência e as proporções das características sociodemográficas e clínicas de SG e SC; e como variáveis independentes, os anos do estudo (2011 a 2022 para a taxa de incidência e 2011 a 2023 para as demais análises). A escolha por esse modelo de regressão de Prais-Winsten deve-se à possibilidade de corrigir a autocorrelação serial proveniente de séries temporais. Para a correção da heterogeneidade da variância dos resíduos transformou-se o desfecho por meio da escala logarítmica.^10^

Posteriormente, foi calculada a variação percentual média anual (AAPC, do inglês average annual percent change?). Para o cálculo da APC, utilizou-se a seguinte fórmula apresentada no estudo de Antunes e Cardoso^10^: APC = (-1+10[b1]*100%), onde o b1 refere-se ao coeficiente angular (beta) da regressão de Prais-Winsten.^10^

Os intervalos de confiança de 95% (IC_95_%) das medidas de APC também foram calculados, segundo a fórmula: IC_95_% mínimo (-1+10[b1-t*e]*100%) e IC_95_% máximo (-1+10[b1+t*e ]*100%).

Os resultados provenientes da regressão foram interpretados da seguinte forma: tendência crescente, quando o coeficiente angular da regressão era positivo e apresentou o valor de p-valor < 0,05; ou tendência decrescente, quando o coeficiente angular da regressão era negativo e o valor de p-valor < 0,05; ou tendência estacionária, quando o valor de p-valor > 0,05.^10^

Por se tratarem de dados públicos não nominais, disponíveis pelo DATASUS, não foi necessária a aprovação do projeto do estudo por um Comitê de Ética em Pesquisa.

## RESULTADOS

No período de 2011 a 2022 foram identificados em São Paulo 125.776 casos de sífilis gestacional e 42.418 casos de sífilis congênita. Nesse período notou-se um aumento nas taxas de incidência de SG e SC (Figura 1). 

**Figura 1:** Taxa de incidência de sífilis gestacional e congênita, São Paulo, 2011-2022

Na Tabela 1 pode-se observar, entre os casos notificados de SG, que 56,43% (n = 70.980) das mulheres encontram-se na faixa etária de 20 a 29 anos, 29,52% (n = 37.132) possuem ensino médio completo e 20,99% (n = 26.397) apresentaram escolaridade ignorada, 41,62% (n = 52.345) declararam-se de cor branca, enquanto 41,07% (n = 51.662) declararam-se de cor parda.

**Tabela 1:** Distribuição absoluta e elativa (%) das características sociodemográficas dos casos de sífilis gestacional e congênita, São Paulo, 2011-2023

**Características sociodemográficas**	**n**	**%**
**Sífilis gestacional**		
**Idade materna (anos) (n = 125.775)**		
10-14	993	0,79
15-19	26.719	21,24
20-29	70.980	56,43
30-39	24.287	19,31
≥ 40	2.796	2,22
**Escolaridade (anos) (n = 125.776)**		
Analfabeta	310	0,25
1ª a 4ª série incompleta	3.433	2,73
4ª série completa	3.258	2,59
5ª a 8ª série incompleta	16.385	13,03
Ensino fundamental completo	12.634	10,04
Ensino médio incompleto	21.913	17,42
Ensino médio completo	37.132	29,52
Ensino superior incompleto	2.213	1,76
Ensino superior completo	2.101	1,67
Ignorado	26.397	20,99
**Raça/cor da pele (n = 125.776)**		
Branca	52.345	41,62
Preta	14.658	11,65
Amarela	730	0,58
Parda	51.662	41,07
Indígena	263	0,21
Ignorado	6.118	4,86
**Sífilis congênita**		
**Idade da criança (n = 38.417)**		
Menos de 7 dias	37.344	97,21
7 a 27 dias	420	1,09
28 a 364 dias	561	1,46
1 ano	55	0,14
2 A 4 anos	26	0,07
5 a 12 anos	11	0,03
**Idade materna (anos) (n = 42.418)**		
10-14	232	0,55
15-19	8.078	19,04
20-29	23.726	55,93
30-39	8.808	20,76
≥ 4	1.007	2,37
Ignorado	567	1,34
**Escolaridade da mãe (n = 42.418)**		
Analfabeta	261	0,62
1ª a 4ª série incompleta	1.350	3,18
4ª série completa	977	2,30
5ª a 8ª série incompleta	6.000	14,14
Ensino fundamental completo	4.549	10,72
Ensino médio incompleto	6.060	14,29
Ensino médio completo	9.844	23,21
Ensino superior incompleto	528	1,24
Ensino superior completo	571	1,35
Não se aplica	216	0,51
Ignorado	12.062	28,44
**Raça/cor da pele da mãe (n = 42.418)**		
Branca	17.300	40,78
Preta	3.485	8,22
Amarela	105	0,25
Parda	18.027	42,50
Indígena	70	0,17
Ignorado	3.431	8,09
**Diagnóstico final (n = 42.418)**		
Sífilis congênita recente	38.110	89,84
Sífilis congênita tardia	38	0,09
Aborto por sífilis	2.703	6,37
Natimorto por sífilis	1.567	3,69

Entre as notificações de SC, 97,21% (n = 37.344) eram com idade de menos tinham menos de 7 dias, 55,29% (n = 23.726) nasceram de mães com idade entre 20 e 29 anos, em 23,21% (n = 9.844) a escolaridade materna era ensino médio completo e em 28,44% (n= 12.062) a escolaridade foi ignorada, em 42,50% (n = 18.027) a cor da pele materna declarada era parda, enquanto em 40,78% (n = 17.300), era branca. Entre os diagnósticos, 89,84% (n = 38.110) eram de sífilis congênita recente.

Com relação às características clínicas, 58,05% (n = 73.010) dos casos de SG eram de sífilis latente e 53,74% (n = 67.594) dos casos de SG foram identificados no 1º trimestre de gestação. Já nos casos de SC, 60,65% (n = 25.727) foram diagnosticados durante o pré-natal da mãe, enquanto 32,78% (n = 13.906) foram diagnosticados no momento do parto/curetagem; 49,50% (n = 20.996) realizaram tratamento inadequado e 34,10% (n = 14.463) não realizaram nenhum tratamento (Tabela 2).

**Tabela 2:** Distribuição absoluta e relativa (%) das características clínicas de detecção da sífilis gestacional e congênita, São Paulo, 2011-2023

**Características clínicas**	**n**	**%**
**Sífilis gestacional (n = 125.776)**		
**Classificação clínica**	216	0,51
Sífilis primária	24.890	19,79
Sífilis secundária	4.502	3,58
Sífilis terciária	7.346	5,84
Sífilis latente	73.010	58,05
Ignorado	16.028	12,74
1º trimestre	67.594	53,74
2º trimestre	30.629	24,35
3º trimestre	23.972	19,06
Ignorado	3.581	2,85
**Sífilis congênita (n = 42.418)**		
**Momento do diagnóstico da sífilis materna**		
Durante o pré-natal	25.727	60,65
No momento do parto/curetagem	13.906	32,78
Após o parto	1.542	3,64
Não realizado	253	0,60
Ignorado	990	2,33
**Esquema de tratamento materno**		
Adequado	2.759	6,50
Inadequado	20.996	49,50
Não realizado	14.463	34,10
Ignorado	4.200	9,90

Na Tabela 3, encontram-se as proporções de SG e SC segundo as variáveis sociodemográficas e clínicas. Observa-se que o ano de 2022 apresentou o maior número de casos notificados para SG e SC. Em relação aos dados sociodemográficos, a idade materna de 20-29 anos predominou durante todos os anos de análise. Quanto ao período de diagnóstico, continuou maior o diagnóstico durante o 1º trimestre para SG. Quanto às características clínicas para diagnóstico, as proporções são maiores para sífilis latente desde o início nos casos de SG e sífilis congênita recente para os casos de SC. Acerca do esquema de tratamento materno nos casos de SC, predominou o tratamento inadequado durante todo o período.

**Tabela 3:** Proporção de sífilis gestacional e congênita segundo variáveis sociodemográficas e clínicas por ano das notificações, São Paulo, 2011-2023

**Variáveis sociodemográficas e clínicas**	**Ano**												
	**2011**	**2012**	**2013**	**2014**	**2015**	**2016**	**2017**	**2018**	**2019**	**2020**	**2021**	**2022**	**2023**
**Caso de sífilis gestacional**													
	n = 3.234	n = 3.899	n = 5.022	n = 6.392	n = 7.138	n = 8.437	n = 10.747	n = 12.467	n = 12.841	n = 14.067	n = 15.317	n = 18.702	n = 7.513
**Proporção dos casos de sífilis gestacional segundo período do diagnóstico**													
1º trimestre	35,56	37,42	39,53	41,96	44,89	49,47	53,24	52,39	53,37	58,51	60,53	61,58	64,09
2º trimestre	32,90	33,24	34,93	32,81	32,56	30,45	27,37	24,27	23,51	20,30	18,92	18,67	17,21
3º trimestre	28,01	26,08	22,66	22,43	19,91	17,71	17,25	20,12	19,54	18,48	17,99	16,86	15,75
Ignorado	3,53	3,26	2,89	2,80	2,65	2,37	2,14	3,22	3,58	2,71	2,57	2,89	2,95
**Proporção dos casos de sífilis gestacional segundo faixa etária (anos) da gestante do momento do diagnóstico**													
10-14	0,87	1,03	1,06	0,97	0,87	1,02	0,81	0,92	0,62	0,84	0,65	0,66	0,53
15-19	18,34	20,24	21,55	22,43	23,26	23,56	23,68	22,53	22,40	22,26	20,51	18,18	16,92
20-29	49,88	49,60	51,19	51,28	52,28	52,74	53,44	54,53	56,86	57,69	59,82	61,87	62,70
30-39	26,50	25,60	23,87	22,48	21,38	20,53	20,01	19,85	18,10	17,20	17,12	17,21	17,66
≥ 40	4,42	3,54	2,33	2,83	2,21	2,15	2,07	2,17	2,02	2,01	1,90	2,07	2,18
**Proporção dos casos de sífilis gestacional segundo a classificação clínica da sífilis gestacional no momento do diagnóstico**													
Sífilis primária	30,12	30,60	26,09	24,78	23,34	22,00	20,94	19,98	16,39	16,19	17,70	17,29	16,49
Sífilis secundária	7,42	6,36	6,39	6,65	5,41	4,39	3,68	3,13	3,03	2,35	2,37	2,37	2,68
Sífilis terciária	10,79	9,05	12,13	11,14	9,53	7,35	6,35	5,57	4,22	4,17	3,96	3,67	2,99
Sífilis latente	32,50	37,47	39,11	42,37	45,15	50,87	55,28	56,63	62,88	68,66	65,55	66,88	66,92
Ignorado	19,17	16,52	16,29	15,07	16,57	15,40	13,76	14,69	13,47	8,63	10,42	9,79	10,91
**Caso de sífilis congênita**													
	n = 1.485	n = 1.919	n = 2.394	n = 3.006	n = 3.450	n = 3.732	n = 4.131	n = 4.041	n = 3.967	n = 3.699	n = 4.004	n = 4.536	n = 2.054
**Proporção do diagnóstico Final**													
Sífilis congênita recente	92,59	91,45	91,85	89,79	90,52	91,56	90,83	88,37	88,73	88,54	88,54	88,93	89,82
Sífilis congênita tardia	0,13	0,21	0,00	0,10	0,12	0,05	0,15	0,02	0,18	0,03	0,02	0,13	0,05
Aborto por sífilis	4,38	4,64	4,76	5,12	5,45	4,88	5,52	7,60	7,71	7,79	7,52	7,67	6,48
Natimorto por sífilis	2,90	3,70	3,38	4,99	3,91	3,51	3,51	4,01	3,38	3,65	3,92	3,26	3,65
**Proporção dos casos de sífilis congênita segundo o esquema de tratamento materno**													
Adequado	3,77	2,92	3,26	2,69	4,09	4,85	4,94	7,13	6,78	8,27	10,06	11,02	9,54
Inadequado	52,32	51,38	51,38	55,59	52,64	55,68	53,84	48,82	46,99	44,93	42,06	44,93	48,34
Não realizado	35,96	38,82	34,00	32,90	31,71	28,91	29,61	33,18	35,44	34,31	41,23	36,18	32,96
Ignorado	7,95	6,88	11,36	8,82	11,57	10,56	11,62	10,86	10,79	12,49	6,64	7,87	9,15

Na Tabela 4, é possível observar os resultados da análise de tendência, bem como a sua APC de taxa de incidência de SG e SC e das proporções dos casos de SG e SC segundo variáveis sociodemográficas e clínicas das notificações. Observou-se tendência crescente nas taxa de incidência de sífilis gestacional (2011-2022) e taxa de incidência de sífilis congênita (2011-2022), além nas proporções dos casos de SG segundo o período gestacional do diagnóstico de sífilis no 1º trimestre (APC = 5,10%; IC_95_% 4,20; 6,00), nas proporções de SG segundo faixa etária da gestante no momento do diagnóstico de sífilis para faixa de 20-29 anos (APC = 1,98%; IC_95_% 1,54; 2,42), nas proporções segundo raça/cor da pele no momento do diagnóstico de sífilis para cor parda (APC = 2,05% - IC_95_% 1,68; 2,41), nas proporções segundo a classificação clínica da SG no momento do diagnóstico de sífilis para sífilis latente (APC = 6,33% - IC_95_% 4,41; 8,30) e nas proporções de SC segundo o esquema de tratamento materno para tratamento adequado (APC = 12,42% - IC_95_% 9,17; 15,76).

**Tabela 4:** Tendência e variação em (%) e média anual com intervalos de confiança de 95% (IC95%)da taxa de incidência de sífilis gestacional e congênita e das proporções dos casos de sífilis gestacional e congênita segundo variáveis sociodemográficas e clínicas das notificações, São Paulo, 2011-2023

Variáveis demográficas e clínicas	% (IC _95_ % )	p-valor	Tendência
**Taxa de incidência de sífilis gestacional (2011-2022)**	**18,68 (16,57; 20,84)**	**< 0,001**	**Crescente**
**Taxa de incidência de sífilis congênita (2011-2022)**	**11,99 (7,00; 17,22)**	**< 0,001**	**Crescente**
**Casos de sífilis gestacional segundo idade gestacional do diagnóstico**			
1º trimestre	5,10 (4,20; 6,00)	< 0,001	Crescente
2º trimestre	-5,62 (-7,42; -3,78)	< 0,001	Decrescente
3º trimestre	-4,20 (-6,15; -2,22)	0,001	Decrescente
Idade gestacional	-0,87 (-3,93; 2,28)	0,554	Estacionária
Ignorada			
**Casos de sífilis gestacional segundo faixa etária (anos) da gestante do momento do diagnóstico**			
10-14	-4,53 (-5,85;- 3,19)	< 0,001	Decrescente
15-19	-0,67 (-3,96; 2,73)	0,670	Estacionária
20-29	1,98 (1,54; 2,42)	< 0,001	Crescente
30-39	-3,47 (-4,62; -2,31)	< 0,001	Decrescente
≥ 40	-5,00 (-8,29; -1,58)	0,009	Decrescente
**Casos de sífilis gestacional segundo a raça/cor da pele no momento do diagnóstico**			
Branca	-1,28 (-1,55; -1,01)	< 0,001	Decrescente
Preta	-0,56 (-1,46; 0,34)	0,203	Estacionária
Amarela	-4,05 (-6,83; -1,19)	0,011	Decrescente
Parda	2,05 (1,68; 2,41)	< 0,001	Crescente
Indígena	-12,92 (-19,05; -6,34)	0,002	Decrescente
Ignorado	-4,25 (-9,14; 0,90)	0,099	Estacionária
**Casos de sífilis gestacional segundo a classificação clínica da sífilis gestacional no momento do diagnóstico**			
Sífilis primária	-5,25 (-6,74; -3,73)	< 0,001	Decrescente
Sífilis secundária	-9,15 (-12,38; -5,80)	< 0,001	Decrescente
Sífilis terciária	-10,88 (-13,61; -8,06)	< 0,001	Decrescente
Sífilis latente	6,33 (4,41; 8,30)	< 0,001	Crescente
Diagnóstico ignorado	-5,30 (-7,10; -3,46)	< 0,001	Decrescente
**Casos de sífilis congênita segundo o esquema de tratamento materno**			
Adequado	12,42 (9,17; 15,76)	< 0,001	Crescente
Inadequado	-1,21 (-2,73; 0,35)	0,118	Estacionária
Não realizado	-0,13 (-2,45; 2,25)	0,910	Estacionária
Ignorado	0,24 (-3,63; 4,26)	0,898	Estacionária

Foi observada tendência decrescente nas proporções dos casos de SG segundo o período gestacional do diagnóstico de sífilis no 2º trimestre (APC = -5,62% - IC_95_% -7,42; -3,78) e 3º trimestre (APC = -4,20% - IC95% -6,15; -2,22), nas proporções de SG segundo faixa etária da gestante no momento do diagnóstico de sífilis para faixas de 10-14 anos (APC = -4,53% - IC_95_% -5,85; -3,19), 30-39 anos (APC = -3,47% - IC_95_% -4,62; -2,31) e ≥ 40 anos (APC = -5,00% - IC_95_% -8,29; -1,58); nas proporções segundo raça/cor da pele no momento do diagnóstico de sífilis para cor branca (APC = -1,28% - IC_95_% -1,55; -1,01), amarela (APC = -4,05% - IC_95_% -6,83; -1,19) e indígena (APC = -12,92% - IC_95_% -19,05; -6,34); e nas proporções segundo a classificação clínica da SG no momento do diagnóstico de sífilis para sífilis primária (APC = -5,25% - IC_95_% -6,74; -3,73), sífilis secundária (APC = -9,15% - IC_95_% -12,38; -5,80) e sífilis terciária (APC = -10,88% - IC_95_% -13,61; -8,06) (Tabela 4).

## DISCUSSÃO

Após análise dos resultados encontrados neste estudo, foi possível verificar uma tendência crescente para o estado de São Paulo nas taxas de incidência de SG e SC. Ressaltaram-se as tendências crescentes nas proporções de SG para o período gestacional do diagnóstico (1º período) e a classificação clínica no momento do diagnóstico (sífilis latente), bem como para a proporção de casos de SC segundo esquema de tratamento materno adequado. Em relação à característica sociodemográfica, observou-se aumento para a idade de 20 a 29 anos e raça/cor da pele autorreferida parda.

Segundo dados do ano de 2022 do IBGE, a população feminina de São Paulo tem como características a faixa etária prevalente entre 30 e 39 anos e a cor autodeclarada como branca (13.768.294), seguida de parda (6.001.139). Os dados deste estudo demonstraram uma prevalência maior de casos de SG em gestantes entre 20 e 29 anos e em mulheres pardas, resultados diferentes da caracterização da população feminina paulistana. Estudos apontam que mulheres pardas e negras sofrem de forma mais expressiva as condições de desigualdades sociais e, por isso, têm menos acesso às práticas de saúde para educação, prevenção e tratamento adequado.^11^

Em relação ao perfil de raça/cor da pele das gestantes diagnosticadas com SG, foi observado que a maior proporção era para mulheres brancas, enquanto a proporção para SC era para mulheres pardas. A incidência da SC decorre do não diagnóstico em período oportuno, o que não permite o tratamento correto para evitar que haja a transmissão intrauterina.^12^ Esse resultado pode estar associado com o fato de que mulheres pretas e pardas no país usualmente estão em populações mais vulneráveis socialmente e que se encontram em regiões em que a APS, por exemplo, não possui uma cobertura tão efetiva e o acesso a serviços de saúde é mais complexo. Deve-se reconhecer também que há um limite na eficácia de políticas públicas brasileiras para saúde materna no que se refere ao enfrentamento de iniquidades étnico-raciais, refletindo em indicadores que expõem o racismo obstétrico.^13^ Nesse sentido, essas mulheres estão em maior vulnerabilidade para a SC, em decorrência do racismo e suas manifestações como um determinante social estrutural que impõe barreiras para o acesso ao diagnóstico e tratamento oportunos.

Além disso, agrava-se nesse cenário a pandemia de covid-19 que contribuiu para o aumento das iniquidades em saúde, deslocando o foco das ações de saúde para o controle da disseminação do coronavírus. Tal rearranjo das organizações de saúde comprometeu o acesso de determinados grupos populacionais à rede de atenção à saúde para outros casos específicos, entre eles o acesso ao pré-natal.^12^ Entretanto, onde as taxas de SC são mais altas deve haver maiores mais investimentos para reduzir ou zerar as taxas de transmissão vertical.^14^

Apesar dos resultados aumentados para tratamentos inadequados ou não realizados durante o pré-natal, a proporção de SC para esquema de tratamento materno adequado mostrou-se com tendência crescente, o que indica que houve um incremento ao longo dos anos para adesão ao tratamento correto em gestantes diagnosticadas com sífilis, porém, mostra uma falha entre o momento em que é feito o tratamento e o parto, como reinfecção sem rastreio. O padrão ouro de tratamento da SG é a aplicação de penicilina G benzatina intramuscular, que é oferecida pela atenção primária. De acordo com o Ministério da Saúde, o esquema de tratamento para gestantes com sífilis depende da classificação clínica da doença, sendo dose única de 2,4 milhões de unidades IM para sífilis primária, secundária e latente recente; e 3 doses de 2,4 milhões de unidades IM com intervalo de uma semana entre elas para sífilis terciária ou latente tardia.^3^ Nesse sentido, a APS tem um papel fundamental no controle da doença e na oferta de tratamento, na medida em que é a porta de entrada dessas pessoas ao serviço de saúde, o local para que seja feito o tratamento adequado e o canal de comunicação nas ações educativas para prevenção de doenças transmissíveis.^15^

O estudo apresentou tendência crescente significativa para proporções de SG para a classificação clínica no momento do diagnóstico, que foi sífilis latente. Sabe-se que quanto mais recente a infecção por sífilis durante a gestação, maior a agressividade para o bebê, mas também é maior a chance de tratamento efetivo. Um dos desafios do diagnóstico de sífilis latente é não saber em que momento ocorreu a infecção e não saber também o período de duração da doença, pois, geralmente, apresenta-se assintomática podendo ser visualizada apenas pelos testes treponêmicos. Visto isso, percebe-se a importância da testagem durante o pré-natal, como é preconizado pelo Ministério da Saúde: testagem no primeiro trimestre, terceiro trimestre e antes do parto. A partir da testagem, é possível reconhecer as gestantes que necessitam de tratamento precoce ou outros encaminhamentos para confirmar o diagnóstico e iniciar o tratamento.^3^ Outro ponto importante a ser considerado é o papel da APS na abordagem às pessoas que planejam engravidar, sendo recomendada a investigação para sífilis, o que impactaria na incidência de SG, uma vez que seria possível tratar a pretendente antes da concepção.^16^ Entretanto, deve-se reconhecer que o planejamento gestacional é um desafio no país por razões como insuficiência de conhecimento acerca de preparo pré-concepcional e indisponibilidade de auxílio para concepção na rotina dos serviços de saúde.^17^

Em 2020, a partir da Portaria nº 120, de 11 de março, da Secretaria Municipal de Saúde de São Paulo, foi estabelecida uma nova designação ao Programa de Saúde da Gestante e do Recém Nascido do Município de São Paulo, como o programa Mãe Paulistana. Nesse programa, os serviços de saúde devem seguir alguns critérios durante o pré-natal para receber uma certificação e, entre eles, está a captação precoce de gestantes (até 12 semanas de gestação) e a percentagem de realização de teste rápido (TR) para sífilis na primeira consulta do pré-natal na UBS, que acaba se tornando um incentivo aos serviços para estabelecer as condutas adequadas no rastreio da sífilis durante a gravidez. As gestantes também têm alguns critérios a serem seguidos para poder receber o enxoval básico do RN e entre eles está a realização de três exames de Sífilis e três exames de HIV, o que permite a detecção precoce e a possibilidade de tratamento oportuno.^18^

Atualmente, o tratamento da parceria não é levado em conta para definir o tratamento adequado após o diagnóstico. Entretanto, a testagem e o tratamento da parceria em casos positivos auxiliam a evitar as reinfecções e as chances de transmissão para o feto. As taxas de transmissão para o feto em locais que não levam em conta o tratamento adequado da parceria também tendem a ser maiores, justamente pela perda do controle devido às reinfecções e chances de falha do tratamento materno.^19^

A cobertura do Sistema Único de Saúde (SUS) no Brasil supera os 80% e as estratégias propostas pelo Ministério da Saúde para prevenção, diagnóstico e tratamento da sífilis durante o pré-natal são bem fundamentadas e de fácil aplicação. Todavia, os resultados deste estudo mostram números que ainda não estão em concordância com o preconizado pelas instituições nacionais e internacionais de saúde para controle dessa infecção.

Ressalta-se que o rastreio de infecções sexualmente transmissíveis como a sífilis é um indicador importante para avaliação da qualidade do pré-natal. Portanto, a diminuição dos casos de SG e SC deve ser abordada como uma condição de saúde pública, considerando que há uma forma eficaz e rápida de rastreio, um tratamento disponível na maioria das localidades e materiais suficientes para educação da população para prevenção.^3,20^

Entre os principais desafios encontrados na APS, a falta de informação tanto dos profissionais quanto das usuárias de saúde está entre os principais motivos da precarização do cuidado em relação à sífilis. Ademais, a dificuldade de acesso às usuárias que se encontram nos grupos de maior vulnerabilidade para infecções sexualmente transmissíveis, como é o caso da sífilis, também é destacado, visto que são essas gestantes que necessitam de maior equidade para o acesso aos serviços ofertados pela rede de atenção à saúde.^20^

Como limitação, pode-se destacar que o estudo se baseou na notificação passiva dos casos identificados de SG e SC no estado de São Paulo, o que acaba dificultando a análise assertiva, pois pode haver casos de subnotificação, que não entram nas estatísticas. Ademais, os resultados podem ser prejudicados pelo fato da atualização periódica dos dados, interferindo também na análise a depender da data de acesso. Não obstante, os dados apresentados e as discussões propostas pelo estudo contribuem para identificação e análise das condições socioestruturais relacionadas à SG e SC, contribuindo para a proposição de estratégias de enfrentamento dessas infecções.

A análise de tendência temporal evidenciou que as taxas de incidência de sífilis gestacional e sífilis congênita apresentaram tendências crescentes significativas, o que se pode relacionar à classificação clínica da sífilis e à ineficiência do tratamento adequado com os diagnósticos tardios. Evidencia-se uma IST com fácil acesso à testagem e identificação da infecção, disponibilidade de tratamento adequado e oportuno, embora haja uma grande quantidade de casos ainda hoje. Com isso, sugere-se uma capacitação continuada dos profissionais de saúde, principalmente da APS, para a identificação da infecção, tratamento correto e apoio às ações educativas de prevenção da sífilis na gestação e agravos para os RNs, com foco principal no eixo pré-natal.

Nesse sentido, faz parte do escopo de atuação da enfermagem as ações de educação em saúde e assistência integral à saúde da mulher, sendo, portanto, uma categoria profissional importante para a prevenção, diagnóstico e cuidado nos casos de SG e SC. No entanto, essas ações devem estar articuladas com outros profissionais de saúde da APS, para que, de forma multidisciplinar, haja o controle das infecções por sífilis na gestação.
